# Financial stability in response to climate change in a northern temperate economy

**DOI:** 10.1038/s41467-021-27490-3

**Published:** 2021-12-09

**Authors:** Kayla Stan, Graham A. Watt, Arturo Sanchez-Azofeifa

**Affiliations:** 1 University of Alberta, Earth and Atmospheric Sciences Department, Centre for Earth Observation Sciences (CEOS), Edmonton, AB T6G 2E3 Canada; 2 Royal Bank of Canada, Innovation & Technology, Climate Solutions, Toronto, ON M5J 0B8 Canada

**Keywords:** Climate-change impacts, Projection and prediction, Business, Projection and prediction

## Abstract

Climate change will have considerable impact on the global economy. Estimates of the economic damages due to climate change have focused on the effect of average temperature, but not the effect of other important climate variables. Related research has not explored the sub-annual economic cycles which may be impacted by climate volatility. To address these deficits, we propose a flexible, non-linear framework which includes a wide range of climate variables to estimate changes in GDP and project sub-annual economic cycle adjustments (period, amplitude, trough depth). We find that the inclusion of a more robust set of climate variables improves model performance by over 20%. Importantly, the improved model predicts an increase in GDP rather than a decrease when only temperature is considered. We also find that climate influences the sub-annual economics of all but one province in Canada. Highest stressed were the Prairie and Atlantic regions. Least stressed was the Southeastern region. Our study advances understanding of the nuances in the relationship between climate change and economic output in Canada. It also provides a method that can be applied to related economies globally to target adaptation and resilience management.

## Abstract (web summary)

Climate change will impact the global economy. Here, the authors propose a framework to evaluate its effect on economies across multiple regional and temporal scales, and project decreased financial stability in a northern temperate economy.

## Introduction

Over the past decade, research has focused on understanding the interaction between climate change and the global economy^1–4^. Shifts in temperature and precipitation patterns and extreme weather events will drive projected economic damages^3,5–7^ and create risks to infrastructure and financial stability^1,8,9^. Climate change has already impacted economic stability: For example, the cost of debt in V20 (Vulnerable 20) countries has increased by $40 billion in the past decade^10^. A critical challenge to financial stability includes climate-induced physical risks, of which the increased severity and frequency of acute events such as hurricanes, floods, droughts, and heatwaves are particularly impactful^11–14^. As a result, there is increased interest from government and financial institutions to incorporate climatic risk into adaptation and resilience management at the local, regional, and national level^14,15^. A focus of those efforts is to identify areas of investment that increase social preparedness and buffer infrastructural burdens due to physical climate risks^14^. Adaptation and resilience management must be heavily targeted at sub-national levels as the impacts felt at local levels are expected to exceed global level trends^15^.

While estimates of economic impacts due to climate change are aligning^1,16,17^, the approaches employed in previous literature focus predominantly on probabilistic modelling and lack attention to critical elements^11,12,18^. Climate-related economics only accounts for a fraction of the existing research, with limited attention to financial stability (Supplementary Fig. 1). Moreover, recent literature has highlighted the need to better understand aggregated economic output in addition to damages^19^. The existing literature in climate-related economics primarily used average temperatures in assessments, with no consideration for other climatic variables that may impact climate-related damage estimates^1,11,12,16,17^. Additional gaps centre on the lack of attention to sub-annual data, a temporal resolution that can capture economic cycle components (e.g., recession timing and trough depths) related to financial stability^1,16,17,20^.

In this work, we outline an empirically based method to project economic changes due to climatic change. We reveal sub-annual economic cycle changes across geographic regions, with particular focus on changes in cycle length, amplitude, and trough depth, as these contribute to financial stability. The performance of common models and importance of a comprehensive suite of climate variables were determined. Canada was chosen to exemplify our method as it is consistently one of the top global economies, one of the most robust, and the fastest growing G7 country prior to disruptions caused by the Coronavirus Pandemic^21,22^. As a northern temperate economy, Canada faces complex climate change caused by disproportionate warming (i.e. warming more than double the global average)^23^ and a longitudinal and latitudinal breadth that experiences a range of climate variability^24,25^. As such, application of our methods to Canada provides a comprehensive example that demonstrates many common challenges faced in economies with less complex climate scenarios. Our study advances understanding of the impacts of climate change on economies and offers insights to guide research and policy development, particularly in other northern temperate economies.

## Results

### Estimated impact of climate change to Canadian GDP

**Fig. 1:**
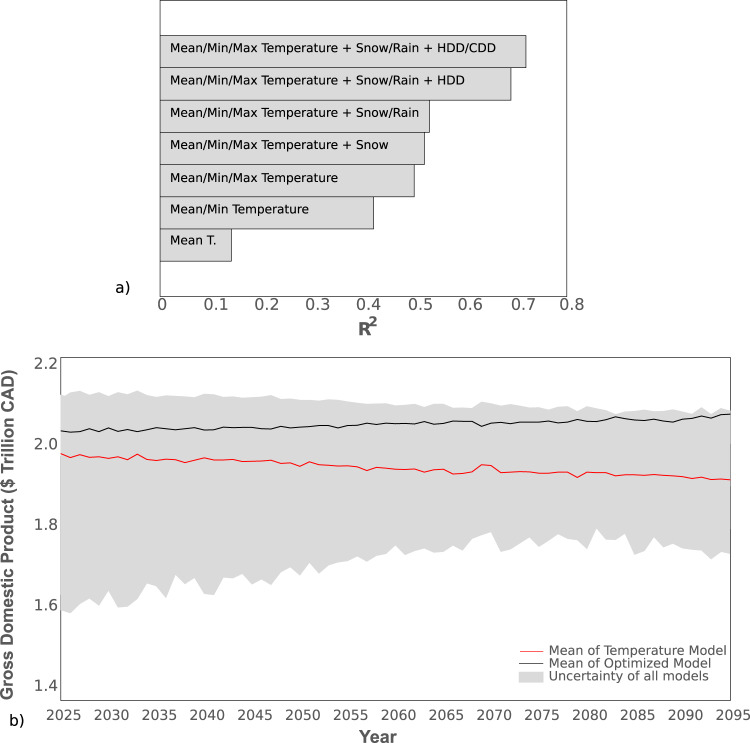
Relationship between climate variables and Canadian gross domestic product (GDP). **a** Accumulated variance explained within the national monthly MARS model. Each bar indicates the *R*^2^ value of a MARS model run according to an incremental increase of one climate variable. **b** Mean Canadian GDP projected from 2025 to 2095 under a high emissions climate scenario (i.e., Representative Concentration Pathway 8.5). Shown are the optimized national model (black), temperature-only model (red), and the range for all other stepped models (grey). GDP does not account for a mean historical annual growth of 2.6% and only indicates the changes based on climatic volatility.

Inclusion of a broad suite of climate variables improves the accuracy of models which attempt to quantify climate−economic relationships. Mean temperature alone explains up to 40% of model variability in Canadian GDP (Fig. 1a). However, the inclusion of precipitation and snow improved the explanation of model variance by 10 % (*R*^2^ to 0.53; GR^2^ = 0.38). Heating and cooling degree days further improved the explanation of variance by 19% (*R*^2^ to 0.72; GR^2^ = 0.5) (Fig. 1a). Quantified economically, annual GDP in 2095 was under-predicted by $400 billion CAD (20%) under the high emissions climate scenario (RCP 8.5) when mean temperature was the only climate variable considered compared to the optimized MARS model (Supplementary Figs. 2 and 3 and Supplementary Table 1).

When applied to the Canadian economy from 2025 to 2095 in a high emission climate change scenario (RCP 8.5), our model projects an annual increase of 0.03% above the normal average GDP growth rate from the past two decades (i.e., 2.6%) (Fig. 1b). Over that 70-year projection period, total annual GDP was estimated to be 2.5% higher than current values based solely on climatic change. When only temperature variables were considered, GDP decreased by 0.04% annually, or 2% by 2095 (Fig. 1b). Moreover, the cumulative difference was projected to be 5%, or $7.4 trillion CAD, over the projection period (Fig. 1b). These values of GDP only indicate changes based on climatic volatility and do not account for the mean historical annual growth of 2.6%.

### Regional economic variability due to climate change

There are four main Canadian regions with similar climatic conditions and economic drivers^26^. These regions include the Pacific Maritime/Western Cordillera, which includes British Columbia, Prairie/Northwestern Forest, which includes Alberta, Saskatchewan, and Manitoba, Southeastern/Northeastern Forest, which includes Ontario and Quebec, and Atlantic Maritime, which includes Nova Scotia, New Brunswick, Newfoundland and Prince Edward Island. The influence of a broad set of climate variables at this regional level was similar to the national level using the MARS model. While mean temperature and precipitation were significant, neither was the most explanatory variable at the regional level (Supplementary Table 2). Cold weather variables, including Snow, Heating Degree Days, and Minimum Temperature, were apex in the majority of regions (Supplementary Table 2). When assessed at a seasonal level, the primary variable of influence varied by province and region, with minimum and maximum temperature the most prevalent in the Prairies, and precipitation and maximum temperature most common in Southeastern Canada. The Atlantic Maritime region was not decomposed into season-specific models.

**Fig. 2:**
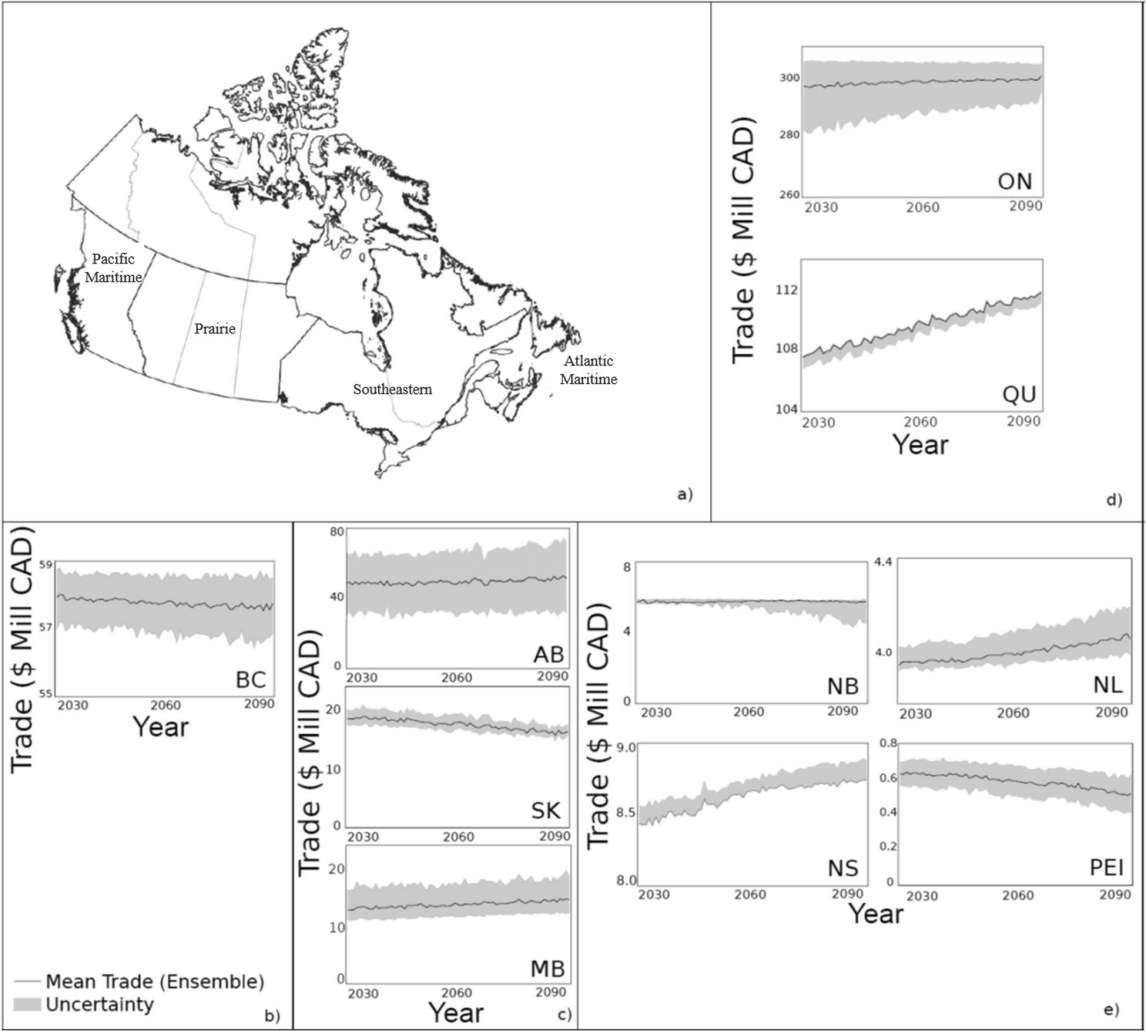
Provincial annual trade projections across regions from 2025 to 2095 under a high emission climate scenario (i.e., Representative Concentration Pathway 8.5). **a** Regions with similar climatic and economic drivers: Pacific Maritime includes British Columbia (BC), Prairie includes Alberta (AB), Saskatchewan (SK), and Manitoba (MB), Southeastern includes Ontario (ON) and Quebec (QC), and Atlantic Maritime includes New Brunswick (NB), Nova Scotia (NS), Newfoundland (NL), and Prince Edward Island (PEI). **b**–**e** Annual trade projected with MARS models optimized by province (black), and the total range of trade projected from all 24 Coupled Model Intercomparison Project 5 data (grey). Projections are shown by region: **b** Pacific Maritime, **c** Prairie, **d** Southeastern, and **e** Atlantic Maritime.

When regional MARS models were projected to 2095, we found that GDP varied by region (Fig. 2a and Supplementary Fig. 4). Most of the variability and the greatest changes in GDP projections were associated with the Prairie region. Projected changes to GDP were on average 3.2%, −12.6%, and 11.4% for Alberta, Saskatchewan, and Manitoba, respectively (Fig. 2b and Supplementary Table 3). Uncertainty due to climate model projections was also high in this region (Fig. 2b–d). Historically, the Southeastern region contributed to the bulk of national GDP (i.e., more than 55%) (Statistics Canada). Economic productivity was projected to increase (*p* < 0.05). GDP in Ontario and Quebec were projected to increase by 1.1% and 3.9%, respectively (Fig. 2c). Projections in the Atlantic Maritime were also highly variable, with provinces showing either significant increases or decreases in GDP (Fig. 2d; Supplementary Fig. 4 and Supplementary Table 3). The Pacific Maritime region (i.e., British Columbia) showed no significant changes to productivity, but also had the mildest projected changes in climate amongst provinces^27^.

### Economic cycle stress due to climate change

**Fig. 3:**
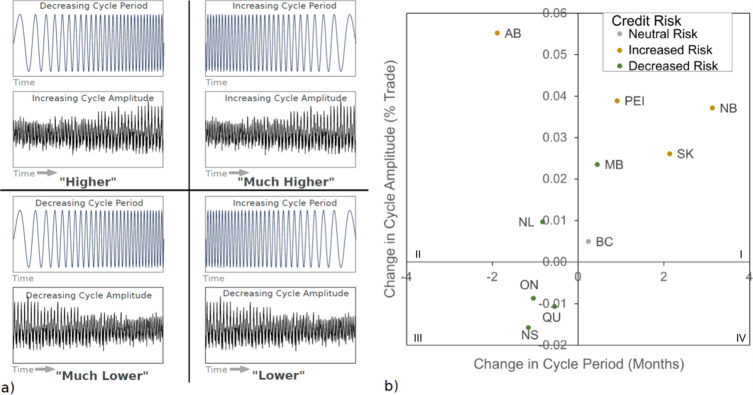
Stress to provincial economic cycles and credit risk in response to climate change from 2025 to 2095 in a high emissions climate scenario (i.e., Representative Concentration Pathway 8.5). **a** Conceptual representation of economic cycle stress according to changes in cycle period (blue) and amplitude (black) by quadrant. Quadrants on the left and right show decreased and increased period, respectively. Top and bottom quadrants show increased and decreased amplitude, respectively. **b** Economic cycle changes and credit risk by province. Changes in amplitude are the percentage change in total trade, while changes in period are in months. Provinces are labelled British Columbia (BC), Alberta (AB), Saskatchewan (SK), Manitoba (MB), Ontario (ON), Quebec (QC), New Brunswick (NB), Nova Scotia (NS), Newfoundland (NL), and Prince Edward Island (PEI). Quadrants are labelled according to categories of stress: (I) Much higher, (II) Higher, (III) Much lower, (IV) Lower. Credit risk is coloured according to change: Neutral (grey), increased (brown) and decreased (green).

In addition to total changes in GDP, we assessed the impact of climate change on the period (i.e., duration of economic season), amplitude (i.e., greatest or least economic output per season), and trend in economic cycles in Canada (Supplementary Table 4). Changes to the economic cycle varied by region. We conceptualized those changes into four categories, and associated quadrants in Fig. 3, of stress to existing economic infrastructure (Fig. 3a): (I) much higher stress (i.e., increased period and amplitude), (II) higher stress (i.e., decreased period and increased amplitude), (III) much lower stress (i.e., decreased period and amplitude) and (IV) lower stress (i.e., increased period and decreased amplitude). In the “much higher” stress quadrant, more economic output, due to favourable climatic conditions, would be required over a longer period. This could mean additional load on infrastructure for an extended duration. In the “higher” stress quadrant, more economic output would be required over a shorter period, again requiring infrastructure to support additional load. In lower stress quadrants, infrastructure would be required to support less load (Fig. 3a).

Provinces estimated to be most stressed by climate change (i.e., greatest change in cycle period and amplitude) included Alberta, Saskatchewan, Prince Edward Island, and New Brunswick. In particular, increased amplitude and decreased period suggest increased volatility, or reduced stability (Fig. 3b and Supplementary Table 4). Those provinces in quadrant III, such as Ontario and Quebec, were estimated to be least impacted with slightly decreased amplitude and period (Fig. 3b). Highly stressed provinces such as Alberta, New Brunswick, Prince Edward Island, and Saskatchewan may experience more extreme trough depth and associated recession severity (Fig. 3b). The opposite is true for Ontario and Quebec given reductions in trough depth.

### Regional economic interdependencies

**Fig. 4:**
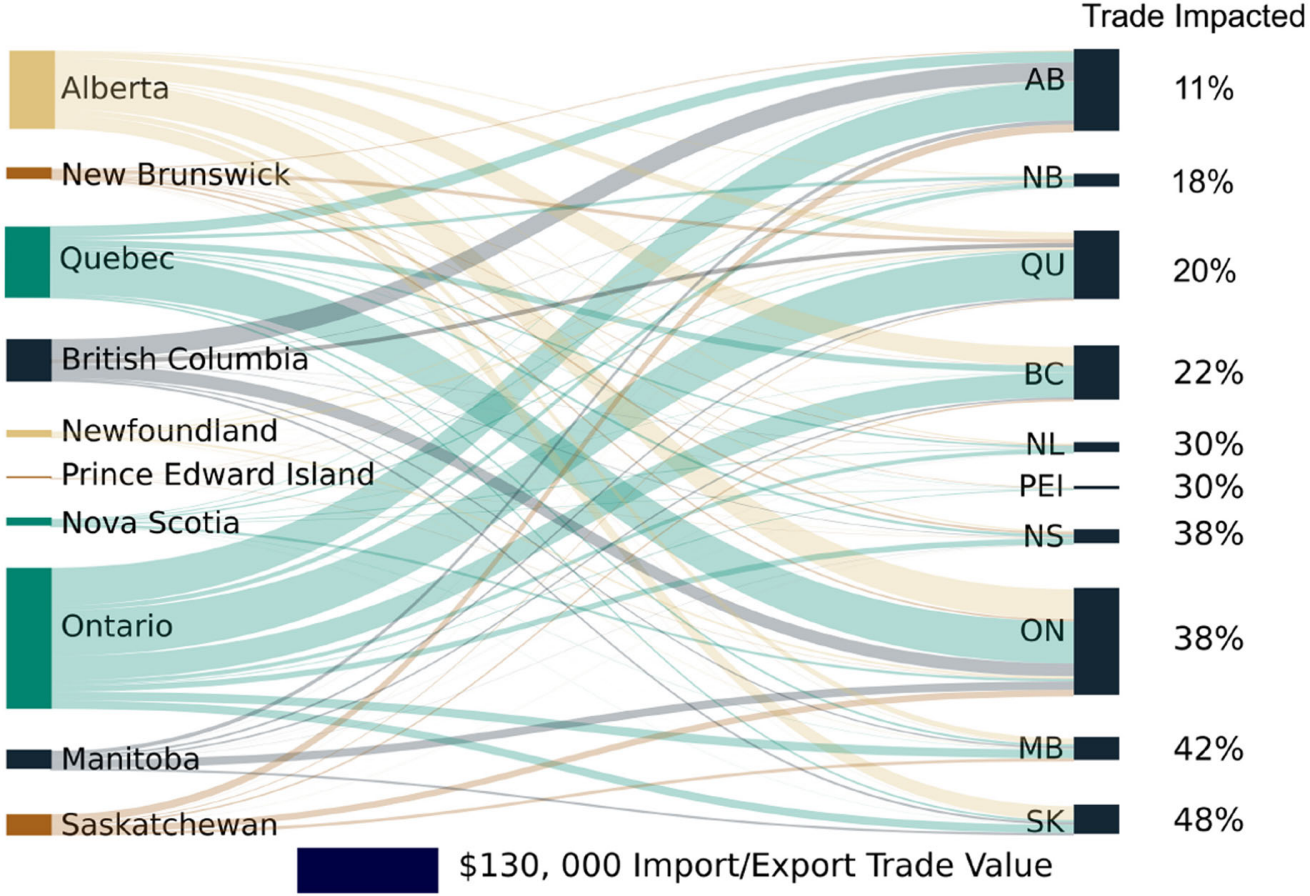
Projected percentage of interprovincial trade impacted by climate between 2025 and 2095 in a high emission climate scenario (i.e., Representative Concentration Pathway 8.5). Provinces coloured green were projected to have increased gross domestic product (GDP) and decreased credit risk, while those coloured yellow were projected to have increased credit risk. Provinces coloured brown were projected to have decreased GDP and increased credit risk. The percentage of imports at risk in each province are listed on the far right.

Interdependency of trade among provinces was the final component of the economic projections in this study. Southeastern Canada was projected to stabilize (based on amplitude, cycle length, and trough depth) with climate change (Fig. 3), which may improve financial stability in other parts of Canada as it is one of the largest trading partners with all other regions (Fig. 4). Saskatchewan’s trade was projected to be most at risk with 48% of its imports derived from high stress zones. Stressed provinces, such as Alberta, Saskatchewan, New Brunswick, and Prince Edward Island, account for an average of 30% of trade across all provinces. Of these provinces, Alberta comprises the majority of the exports, indicating that resilience and adaptive management policies should focus on this province to mitigate climate-related damages.

## Discussion

Other models in the literature that estimate total GDP impact due to climate change have been criticized for not effectively resolving the contribution of cold weather variables^16,17,28^. Such models generally show GDP to decrease^17^, or increase minimally^11^. Our model shows similar trends, but only when temperature was considered alone. We found that a range of explanatory climate variables were both significant in our model and improved performance by 20%. When the range of variables were considered, our model no longer predicted decreased GDP, but rather increased GDP. The increase was 2% greater than other studies that showed short-term GDP growth^16,17^. Our results indicate that using temperature alone as an explanatory variable in estimating the relationship between climate and the economy may be misleading. Our findings instead show that inclusion of a range of explanatory climate variables can improve model performance. Additionally, our study differs from other studies in its sub-annual assessment of economic cycles, where trends are typically presented with annual or climate normal time-scales^11,12,16,17^.

Differences among economic projections can be related to industries that dominate the regional economy. The major industry in the Atlantic Maritime is the fishing industry, and mining in Newfoundland^26^. In the Southeastern region, shipping, manufacturing, international business, and technology development are primary industries^26^. The Prairies is a resource-based economy and the Pacific Maritime is focused on real-estate, shipping, and finance^26^. Understanding climate impacts on regional economies can assist governments in crafting regionally relevant and nationally cohesive economic policies.

Economic cycling, or the annual and sub-annual fluctuations of GDP during periods of growth and recession^29^, is foundational to economic planning and development. For example, investment in infrastructure typically occurs during periods of growth rather than recession^30^. Insufficient investment has caused bottlenecks and delayed development, resulting in reduced economic productivity^30^. Moreover, shocks to the economic cycle, for example due to severe climatic events, can cause stress to infrastructure that requires emergency repair and ongoing investment to improve resiliency^31^. Recent examples of climatic shocks include the 2016 wildfire that destroyed much of Fort McMurray, Alberta, displaced 88,000 people and negatively impacted the economy^32^, or the 2013 Alberta flood, which was the costliest Canadian natural disaster at over $5 billion in insurance claims^33^. Critical components of the economic cycle, such as duration, amplitude (difference between peak and trough economic values over time) and trough depth, can be used to inform such infrastructure investment^29^.

Economic cycle period reduces in both Ontario and Quebec, indicating a stabilization in the economy and reduced credit risk^34^. Given that the Southeastern region is the industrial centre of the country, this increased stability supports stability at the national level. By contrast, highly stressed provinces in the Prairies and Atlantic Maritime are predicted to have increased credit risk, and considerable changes to the amplitude and length of the economic cycle (Fig. 3b), destabilizing the economy and increasing climate-related damages^35^. Increased cycle amplitude stresses infrastructure because it increases the difference between growth and recession cycles. One additional problem is that even though provinces such as Alberta have quickly recovered from historical shocks, the persistence of climate change may prove more difficult to absorb^36^.

Challenges to infrastructure may limit the extent to which a region can capture the economic value created by favourable climatic conditions. This can be due to increases in production or increased amplitude or volatility where opportunity of production increases beyond the limits for which infrastructure is designed, creating physical limitations^30^. For example, where economic load extends beyond the normal range, or over a shortened period, infrastructure may deteriorate faster or fail^37^. Additional load may exceed the capacity of existing infrastructure, causing bottlenecks^30^. If the economic cycle is shortened, the same amount of productivity is required over a shorter timeframe (i.e., supply chains must support the movement of more materials in less time), bottlenecking the current infrastructure and stressing labour. Targeted investment could improve resiliency and shorten recessions^38^. Increases in cycle trough, or the recession portion of the cycle, will further stress seasonal workforces, potentially leading to migration and job losses^31,39^. A decrease in amplitude would result in economic stabilization, improving labour prospects and may incentivizes investment and additional attention to resiliency development^34,40^.

Given the projected changes to economic cycle, we suggest that Alberta and New Brunswick will incur the most extensive stress to infrastructure. Alberta was found to have the greatest increase in amplitude, while New Brunswick the greatest increase in period length. These provinces have predominantly goods-producing economies which are less resilient to economic shocks, and even small shocks can translate into long-term impacts on the economy^36,41–43^. At the national level, high diversity among economic regions in Canada^26^ may contribute more to resiliency than diversification within regions^44^. It should also be noted that there are a number of industries which may individually contribute to increases in economic production and resiliency^45,46^. The wine industry in the Southeastern region is projected to increase in production so long as it pivots to include heat tolerant grape varieties, because fewer crops will be lost to cold weather as minimum temperatures continue to climb^45^. Additionally, more favourable climatic conditions are projected to increase general tourism and recreation activities in Canada, though cold weather tourism may drop with fewer cold weather days^46^. Nonetheless, regions and provinces with higher likelihood of stressed infrastructure should be the focus of capital mobilization and deployment. Such focus could facilitate the capture of new economic potential from areas with economically favourable climate change projections. Without such focus, the benefits of climate change may not be realized, and national economic growth and stability challenged.

Certain caveats should be considered in our findings. Our projections were developed from the historical relationship between climate and the economy. The projections show economic conditions given future climate configurations only, and do not consider changes to other contributing factors (i.e., future conditions consider all else, besides climate, equal). We assume that the global economic system will remain intact. Destabilizing factors related to climate such as, for example, conflict, political turmoil, mass migration, biodiversity loss, food and water shortage, or over population were not considered. Moreover, damages from acute climate-related events such as wildfire, flood, sea level rise, and extreme heat were also not explicitly considered. Our findings may overestimate economic growth given the non-linearity in the relationships between extreme weather and economic damages and that future climate extremes may exceed historical frequency and range. We also modelled economic productivity from the climate conditions projected under RCP 8.5, a high emissions or extreme scenario with a likelihood of occurrence of 35%. Applying our methods to other scenarios, such as RCP 4.5, may provide a more conservative estimate. Further research should consider these factors to provide a more robust estimate of economic impacts in climate scenarios.

Beyond the global geo-political connectedness, another area that is necessary for future research is the expansion of interdependent trade-climate predictions. We show the considerable interdependency between regions, however, research into trade networks and climate change is limited. To better articulate climate−economic interactions and outcomes, future research should explore economic productivity by industry given climate change, regional resilience, and the trade and investment networks that connect the global economy^44,47^.

In conclusion, we outline an empirically robust method that evaluates the effect of additional climate variables on economic projections under climatic change, with focus on the impacts to financial stability based on sub-annual climate analyses. Our method enables the assessment of impacts to economic cycles and the implications for credit risk and stress to infrastructure. We show that economic trends tend to group by climatic and economic regions, which is likely due to similar economic and climatic drivers. Increased stability and reductions in economic cycle amplitude, improved credit risk and increased total trade were estimated for the economic centres of Ontario and Quebec. Conversely, the Prairie region, particularly the provinces of Alberta and Saskatchewan, were projected to be the highest stressed. We highlight the importance of considering a more comprehensive set of climate variables and sub-annual economic trends in estimating the economic impact of climate change. Our method can be applied to other northern temperate economies to expand the dimensionality of the impacts of climate change.

## Methods

### Climate and economic data

The historical provincial climate variables collected included monthly average temperature, maximum temperature, minimum temperature, precipitation (rain), snow, heating degree days, and cooling degree days. These data were all available through the monthly climate summaries distributed by the Government of Canada through the Digital Archive of Canadian Climatological Data (https://climate.weather.gc.ca/prods_servs/cdn_climate_summary_e.html). Future climate data were compiled from the Climate Atlas of Canada and Climate Data Canada portals (https://climateatlas.ca/find-local-data; https://climatedata.ca/download/). Both portals use data provided from the Pacific Climate Impacts Consortium downscaled regional model^48,49^. The data have been downscaled to 10 km resolution using bias correction based on constructed analogues and quantiles^48,49^.

This study uses RCP 8.5 climate data. Those data are considered to be highly relevant for mid-century carbon scenarios and is still highly plausible by the end of the century^50^. While this scenario has been misrepresented in other research as the business-as-usual scenario, it remains a useful scenario as it is congruent with policies and emission projections under those policies to 2050. Given the fluidity of policies over decades, it is difficult to suggest that any scenario will fully capture the trajectory of emissions that will occur over the next century; however, this scenario is projected to have 35% probability of the 8.5 emissions being true until 2100, and even with policy changes^50–52^. The RCP 8.5 scenario is useful for risk assessments^50^. The RCP 8.5 scenario will project the most extreme case of physical and economic risks, allowing for policies to be built with the understanding that damages can be mitigated by continuing to improve policies which buffer against damages and risks^50,51^.

Snowfall data were collected from the CANESM2 model (https://climate-scenarios.canada.ca/?page=pred-canesm2), which is the second-generation earth system model created by the Canadian Centre for Climate Modelling and Analysis and is part of the World Climate Research Project. The historical and future climate data were aggregated to a monthly and provincial scale for consistency across all data. The historical climate data are taken from 2000 to 2019, and the future data range from 2025 to 2095. These dates match the economic and climate data availability while also providing a buffer for economic recovery and stabilization after the coronavirus pandemic.

National GDP data are available through Statistics Canada as part of the Gross Domestic Product by Industry monthly national survey (https://www150.statcan.gc.ca/t1/tbl1/en/tv.action?pid=3610043402). The information is collected at basic prices and includes the costs associated with consumption and capital assets depreciation. These data are used to supplement the annual national and provincial GDP Supply and Use tables collected for this study (https://www150.statcan.gc.ca/n1/pub/15-602-x/15-602-x2017001-eng.htm). These data are also provided through Statistics Canada; however, they are an annual measure of the economic activity undertaken in the country based on the production of goods and services, the final sale of these goods and services, and any supplementary financial transactions. These data are insufficient to produce provincial economic cycling accounts, and therefore additional trade data are required. Monthly provincial wholesale trade data are compiled from the Monthly Wholesale Trade Survey provided by Statistics Canada (https://www23.statcan.gc.ca/imdb/p2SV.pl?Function=getSurvey&Id=1305485). This monthly survey is designed to indicate Canadian economic health by providing wholesale merchant monthly sales and inventory levels in each province. By definitions from Statistics Canada, the trade data are closely related to GDP, accounting for roughly 5–6% of the Canadian annual GDP. To generate interconnectivity assessments between the provinces, the interprovincial and international trade flows summary levels from Statistics Canada were used (10.25318/1210008801-eng). These data are collected at an annual level using the Supply Use Input-Output Tables which are used to trace productivity, imports, exports, and final consumption to measure the value of industries at a provincial level. All these datasets are available for the past 20 years in Canada.

### Data cleaning

The economic data were detrended using the average annual growth rate to assess only the impact of climate data instead of including the overall GDP growth rate that Canada naturally experiences. The data were then pooled to reduce the pseudo-replication that would otherwise bias any relationship built between climate and the economy. This commonly used method of reducing pseudo-replication was also run through iterative models at different binning levels and with maximum and minimum values to better characterize the uncertainty of the data pool^53–55^. The data in this study were pooled by temperature and precipitation. For example, Alberta months with an average temperature between 1 °C and 2 °C are averaged together. The data were binned at different levels to test the model and binning sensitivity. For temperature, the data were binned into 1 °C, 2 °C, 3 °C, and 5 °C, and precipitation is binned at 5 mm.

Climate data were also cleaned for any quality control flags, and any flagged data were removed from the analysis. All data were tested for normality using the Shapiro−Wilks test to determine if parametric models could be used^56^. All data were found to be normal using the Shapiro−Wilks test.

### Historical model testing

A series of linear and non-linear, parametric, and non-parametric models were built to test the relationship between climate and the economy. The linear models, tested and compared for significance and *R*^2^, include general linear, generalized linear mixed-effects, and least angle regression models. The generalized linear model is a simple linear regression that uses all variables^57^. The generalized linear mixed-effects model extends normal linear models by including random and fixed effects^58^. Random effects help researchers to parameterize non-independent data and modify fixed effects^59^. The least angle regression model was used to improve the linear model by producing an automatic iterative selection of the best climate variables most highly correlated with the economic data^60^. This model combines the highly aggressive forward stepwise approach and the forward stagewise approach^60^. Forward stepwise models gain the best fit by predicting the best fit variable and then calculating the residuals and adding the variable which best removes those residuals^60,61^. Forward stagewise instead adjusts the relationships in thousands of small incremental steps to find the best fit^59,61^. The LARS model combines forward stepwise and stagewise approaches to reduce the overfitting tendency of the first and the high computational power required for the second^59^.

The non-linear models tested include decision trees, random forest, and multivariate adaptive regression splines. Decision trees test numeric datasets against threshold values, with part of the data used to train the model and the remaining data used to validate the model^62^. Decision trees provide a machine-learning approach that allows for non-parametric data and only include the most important variables^63^. Random Forest is a bagging ensemble approach that extends the decision tree methodology^64,65^. Random forests take points that were misclassified in the original decision tree and creates a new tree to ensure that these points are correctly classified a second time^65^. This process is repeated until an allotted number of trees is created. The forest then uses majority voting to determine the final value^65^. For this study, the random forest contained 1000 decision trees.

The non-linear multivariate adaptive regression spline model used in this study is a piecewise non-linear model which allows for the selection of the best variables to be used and provides the non-linear hinge points^66,67^. This model also allows for interaction between different variables so that if there are covariates that relate, they can be included in a non-linear fashion^67^. This model also permits non-linearly related variables, while others may be linearly related to the output^67^. This combination of factors allows for a broader mixed approach than the previous linear and non-linear models^67^. MARS models have been used in other environmental studies and are as effective as support vector machines and are more effective for estimating peak values than other models, including LSSVM and M5Tree^68^. The MARS model was assessed using both the *R*^2^ and the GR^2^. The GR^2^ was added for the MARS model as it is listed as an estimate of the accuracy, with negative values indicating a heavily over parameterized model^69^. While econometric models often use an F-statistic as part of their accuracy assessments, MARS model do not use this statistic, and the GR^2^ was used as a cross validation substitute^69^.

The optimized model was found by comparing the results of using the different binning techniques, variable inclusion and exclusion, and the model type. The optimal model was determined by the significance levels, *R*^2^, and GR^2^ values to determine which model was optimal (see Supplementary Information). Variable importance was calculated in the optimized model using variable inclusion and exclusion and again by using the stepwise approach which automates optimal variable selection (Supplementary Fig. 2). The optimized model for each province was selected for running the future projections.

### Optimal model selection and variable importance

When Canadian trade data are assessed against climate variables, we find that a non-linear approach explains the most variability. The Least Angle Regression model (LARS), which is a stepwise linear model, was the only linear model tested that showed a significant relationship with climate variables, but unfortunately, it only accounts for a maximum of 0.5 of the variability in any province (Supplementary Fig. 1; *R*^2^ = 0.5). The non-linear models tested included Random Forests (run annually and monthly) and the Multivariate Adaptive Regression Splines (MARS) model. The Random Forest model is highly variable, with Alberta and Saskatchewan performing the worst. New Brunswick does perform well with the monthly Random Forest, but no other provinces reach an *R*^2^ of 0.7 (Supplementary Fig. 1). The MARS model performs much better than the linear models and the random forest. Some provinces, including British Columbia, Alberta, and New Brunswick, perform best when binned at 1 °C, while Manitoba, Quebec, Nova Scotia, and Prince Edward Island perform best with 2 °C binning. Three provinces also perform better when the months are included in the MARS model. These provinces are Saskatchewan, Ontario, and Newfoundland. Overall, six provinces have an *R*^2^ over 0.8, two provinces have an *R*^2^ over 0.7, and the remaining two provinces, Alberta and Nova Scotia, have an *R*^2^ over 0.5 (Supplementary Fig. 1).

With the optimized MARS model by province, we find that eight out of ten provinces have at least one cold-weather variable as a critical variable to predict the economic variability (Supplementary Fig. 2). The cold weather variables are Minimum Temperature, Snow, and Heating Degree Days. Alberta and Newfoundland are the only exceptions, though Newfoundland is optimized when December is used as a variable (Supplementary Fig. 2). Alberta’s most important variables include maximum temperature and precipitation. When monthly data are further restricted to quarters—based on the Statistics Canada quarters, different variables become more critical. During the fall, temperature variables are the most important, with minimum temperature most important for British Columbia and Alberta, maximum temperature most important for the Central provinces of Saskatchewan, Manitoba, Ontario, and Quebec. The average temperature is most important for New Brunswick, and the remaining Atlantic provinces were not able to be predicted using just only data from fall. Minimum and maximum temperature, along with precipitation, are most important in winter. The maximum temperature was most valuable in BC and Alberta, the minimum temperature for Saskatchewan and Quebec, and rain for Manitoba and Ontario. The Atlantic provinces were unable to have the economic variables predicted using climate data only from winter. In Spring and Summer, the most important variables are less consistent. Only Newfoundland and Nova Scotia are unable to have trade predicted by climate.

### Future models and uncertainty assessments

Using the optimized pooling and MARS model construction for each province, future scenarios were built using the Climate Atlas Data. These data were modelled from 2025 to 2095 as that is the timeframe with data for all variables. Additionally, this timeframe allows for the recovery of the economy from the coronavirus pandemic to new steady-state conditions so that this extraneous event will not influence the relationship found between weather and the economy. The economic values were projected at a monthly scale for each province. The mean economic value from the binning was used, as well as the minimum and maximum. The climate data from each of the 24 models were projected forward using the mean economic value, in addition to the Ensemble, 10th percentile, and 90th percentile climate model data.

The monthly and yearly projections were assessed for trends using the Mann−Kendall and seasonal Mann−Kendall statistical tests. These tests are a non-parametric statistic that has historically been used in hydrometeorological time-series studies^38,70–72^. These statistics were tested for significance and the directionality of the trend.

The uncertainty of future predictions is assessed on two fronts. The first uncertainty assessment accounts for the variation in the binning process (Supplementary Table 1). The models built from the mean, minimum, and maximum economic values in the binning were projected. Due to the models’ non-linear nature, the uncertainty is non-symmetrical but does test the sensitivity of the modelling and binning process. The second uncertainty assessment is found from running the mean economic value using the 27 climate models. This uncertainty was calculated at the monthly and annual levels.

### Economic cycling

The monthly data allow for the economic cycles to be assessed for the length of the cycle, peak values, trough values, and overall variability^38^. Classically, time-series assessments of business cycles must have at least 15 months of data, meaning that our historical assessments with over 10 years of data and the projections with over 70 years of data are sufficient to determine the parameters^73^. The time-series noise needs to be smoothed using the Savitsky−Golay filter in the TIMESAT program to determine the features of interest^74^. The Savitsky−Golay filter was initially designed to smooth noisy spectral signatures but was expanded to smooth environmental time-series and other cycle-based patterns^75^. TIMESAT was developed initially to assess seasonal patterns in satellite data; however, it can analyse any cyclical data patterns^75^. The start and end of the season parameters were set to 0.25 of peak economic value. Outputs from the TIMESAT analysis include the start, end, and length of the cycle, the peak and trough values, and overall amplitude of the cycle^75^. Mann−Kendall statistics were run on the TIMESAT outputs pertinent for evaluating the economic cycle (seasonality, amplitude, and trough value/ SI4).

Changes to the economic cycle were then plotted on a two-by-two matrix to assess the relative changes to economic cycle length and amplitude. Decreased economic cycle length and increased cycle amplitude relate strongly to increased volatility in the economic cycle and therefore are considered to stress infrastructure the most. The second highest stress category is an increased cycle length and increased amplitude. The increased amplitude creates the highest stress on infrastructure, as the requirements for labour, capital, and physical infrastructure will have larger changes between growth and recession segments of the economic cycle. The reduction in amplitude relates strongly to less stress on infrastructure, and no change to economic cycle also strongly relates to less infrastructure stress the current infrastructure can handle the current bottlenecks and challenges associated with the cycle parameters. Finally, trends in the troughs were used as a proxy for credit risk. The troughs that deepened over time have an increased credit risk, and those that had a reduction in the depth of the trough have a decreased credit risk.

### Trade and GDP relationship

The role of trade on economic growth and development has been a topic of research for many decades, with recent studies finding the most significant impact of trade on economic growth in developed and developing countries^76^. Despite a focus on quantifying trade and economic development, there has been little research on the direct relationship between trade and GDP over longer time scales^77^. The primary body of work in this field instead focuses on the impact of trade policies such as free trade and protectionism^77^. This relationship is essential to quantify; however, when looking towards modelling the interaction between climate and the economy. With a strong relationship between GDP and trade, the ability to build relationships in other countries is extended because there are some countries that have better resolution trade data.

The value of trade only represents a small portion of the economy relating to 5–6% of the gross domestic products, according to Statistics Canada. There is no monthly trade data available at less than a national level; therefore, it was essential to find a relationship between trade and the GDP. This relationship was built in two different ways. The first was to create a correlation and regression between Trade and GDP. The second was to develop a national monthly model between the climate variables and GDP, as well as with trade, to compare the climate variables that were of importance and the hinge point to determine if the GDP values were impacted in the same way at all points by the climate variables. Finally, to assess the impact of the number of climate variables on the GDP, the optimized non-linear model was run with a stepwise approach. The *R*^2^ value was then evaluated to determine how adding different variables impacted the national economic variability.

Overall, there is a robust and significant relationship between the log-transformed trade and the base GDP data (Supplementary Fig. 5; *p* < 0.001; *R*^2^ > 0.9). The strength of the correlation between trade and GDP expands the number of countries with sufficient data to produce climate-econometric research. Additionally, it allows for more comprehensive studies, including trade and investment networks to be built between different countries, and each country’s trade data can be related to climate.

Additionally, trade interconnectivity was assessed based on the Supply Input-Output Tables. The percentage of imports and exports to each country was based on the 5-year average values. To then determine the amount of trade that is at risk of stress, the province with high stress due to decreases in GDP or increased credit risk were identified. The amount of trade that was then imported into a province from these at-risk areas was totalled and compared against the total value of imports. While this analysis is relatively simple, it provides a useful starting point to begin understanding trade relationships and the secondary impacts of climatic damages.

### Reporting summary

Further information on research design is available in the Nature Research Reporting Summary linked to this article.

## Supplementary information

Supplementary Information

Reporting Summary

## Data Availability

The projected trade data generated in this study have been deposited in the Harvard dataverse under accession code 10.7910/DVN/AXGDJO. The binned climate data are available in Harvard dataverse under accession code 10.7910/DVN/TNXUUN. Select monthly climate data are available in the Harvard dataverse under accession code 10.7910/DVN/J0B3QD.
