# Supplementary material for: Financial stability in response to climate change in a northern temperate economy
Source: Nat Commun. 2021 Dec 9;12:7161. doi: 10.1038/s41467-021-27490-3 (PMC8660787; doi:10.1038/s41467-021-27490-3)
Supplement: Supplementary file 1 — Supplementary Information [file 41467_2021_27490_MOESM1_ESM.pdf]

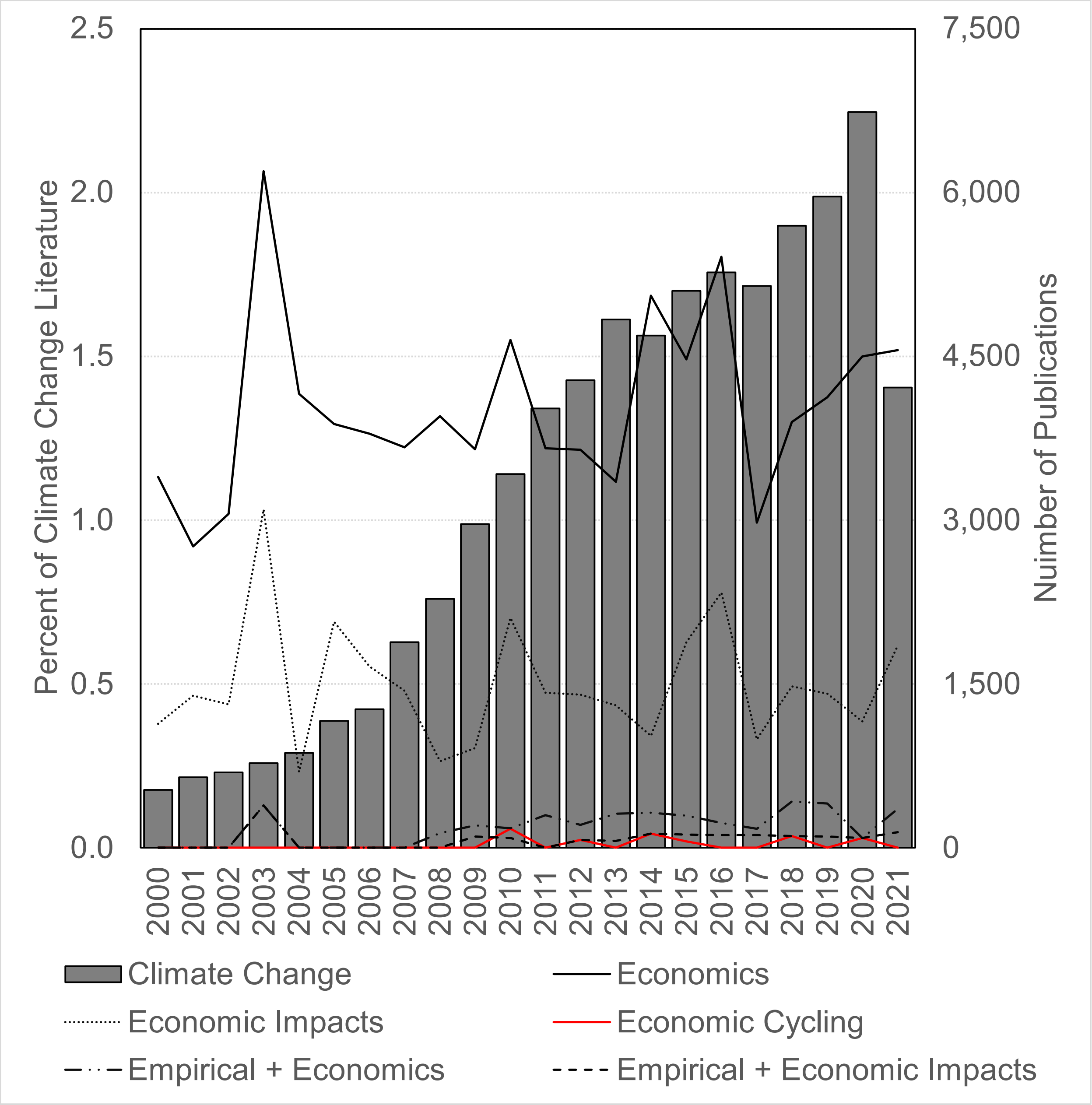

Supplementary Figure 1. Over the past two-decade climate research has increased from approximately 500 papers per year to 6,500. Of this research, economic impacts, cycling, and empirical research comprises a small component of the work being done. This data was pulled from Scopus title and keyword searches.

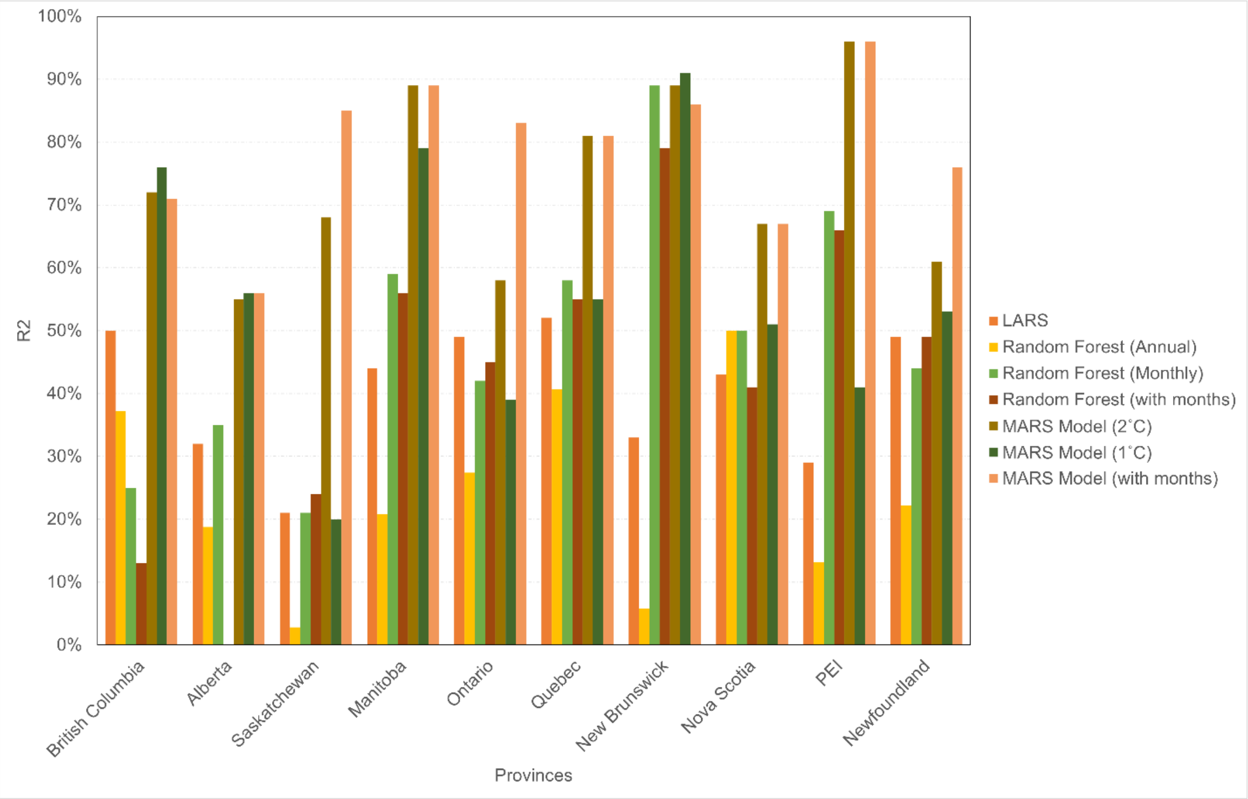

Supplementary Figure 2. The explanation of variance for each of the climate-trade models by province. The LARS model is the only significant linear model and is thus the only linear model represented here. The Random Forest model is divided into three categories – annual trade/climate variables, monthly data, and monthly data that includes which months are found in the bin. The MARS model is the optimal non-linear model, which explains the most variance in all provinces. There is different binning to determine sensitivity with binning by 2˚C, 1˚C, and the optimised binning of each month with the addition of the months being coded into the model.

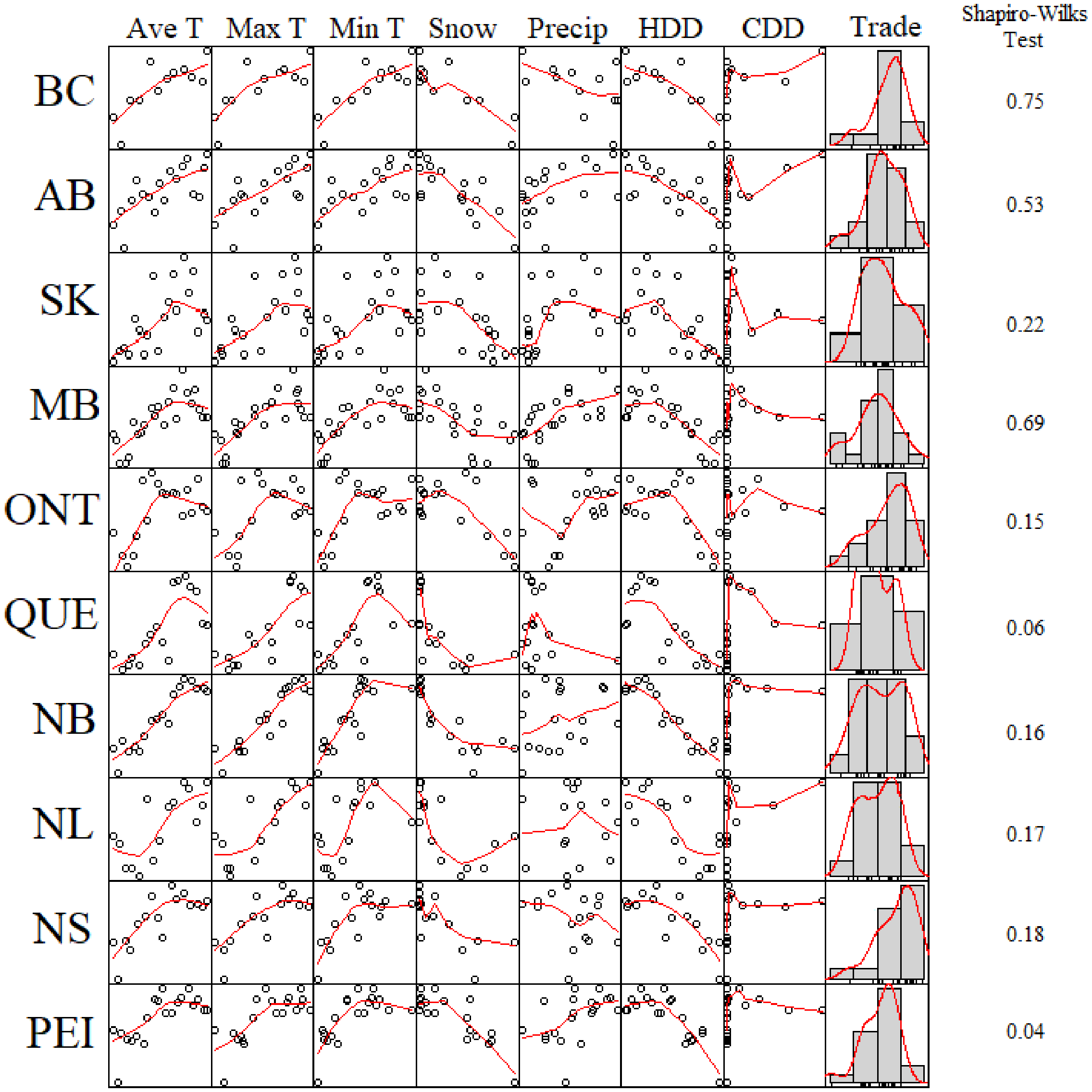

Supplementary Figure 3: The correlation matrix and normality testing (Shapiro-Wilks Statistical Test) for provincial trade data. The correlation matrix shows the data distribution and the correlative relationship between trade and the individual climate variables.

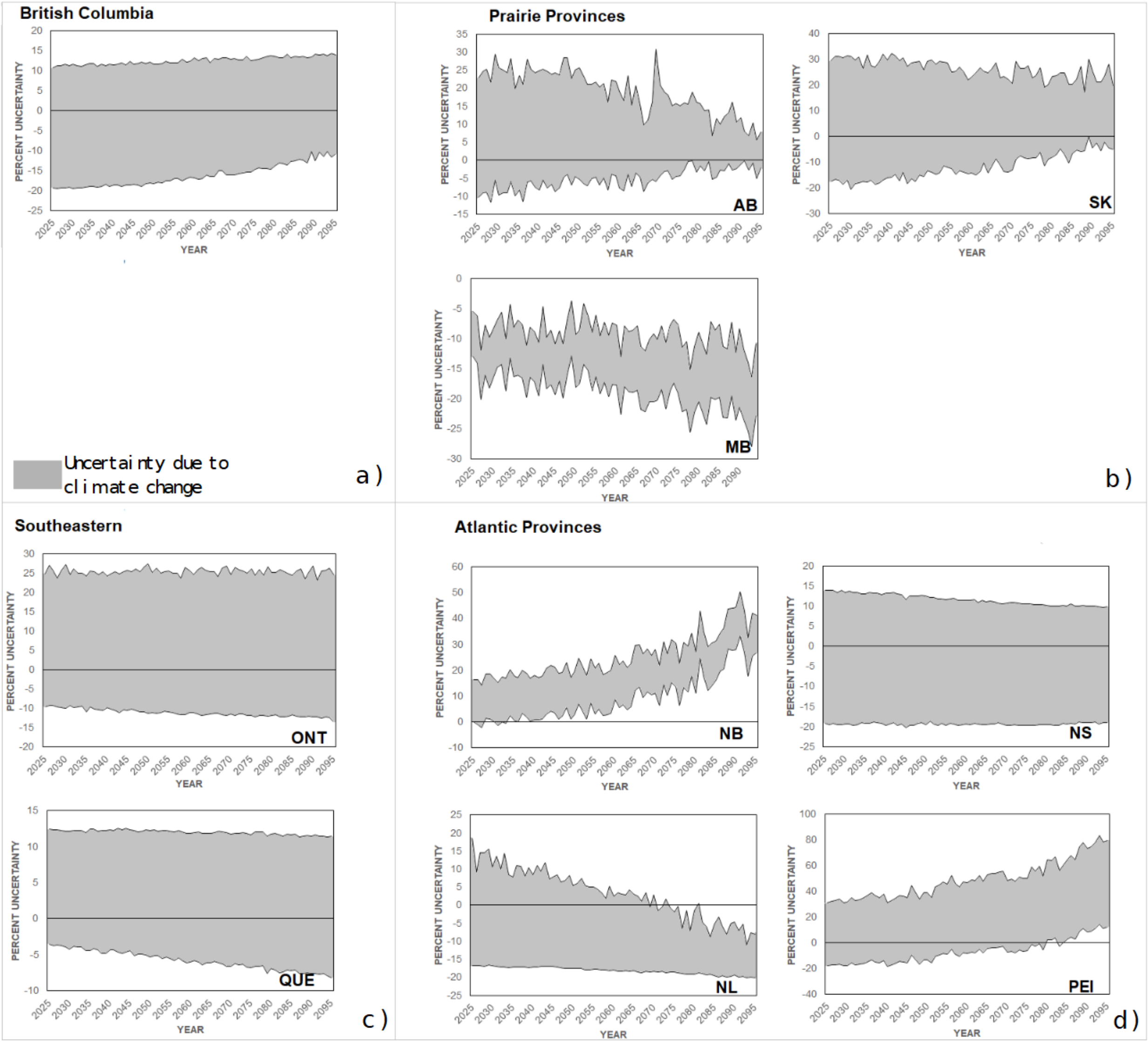

Supplementary Figure 4. The economic uncertainty established by each province using the minimum and maximum binning data. The values are a percentage of the trade value for that year as established by the averaged optimised model. Data are presented as mean values and uncertainty.

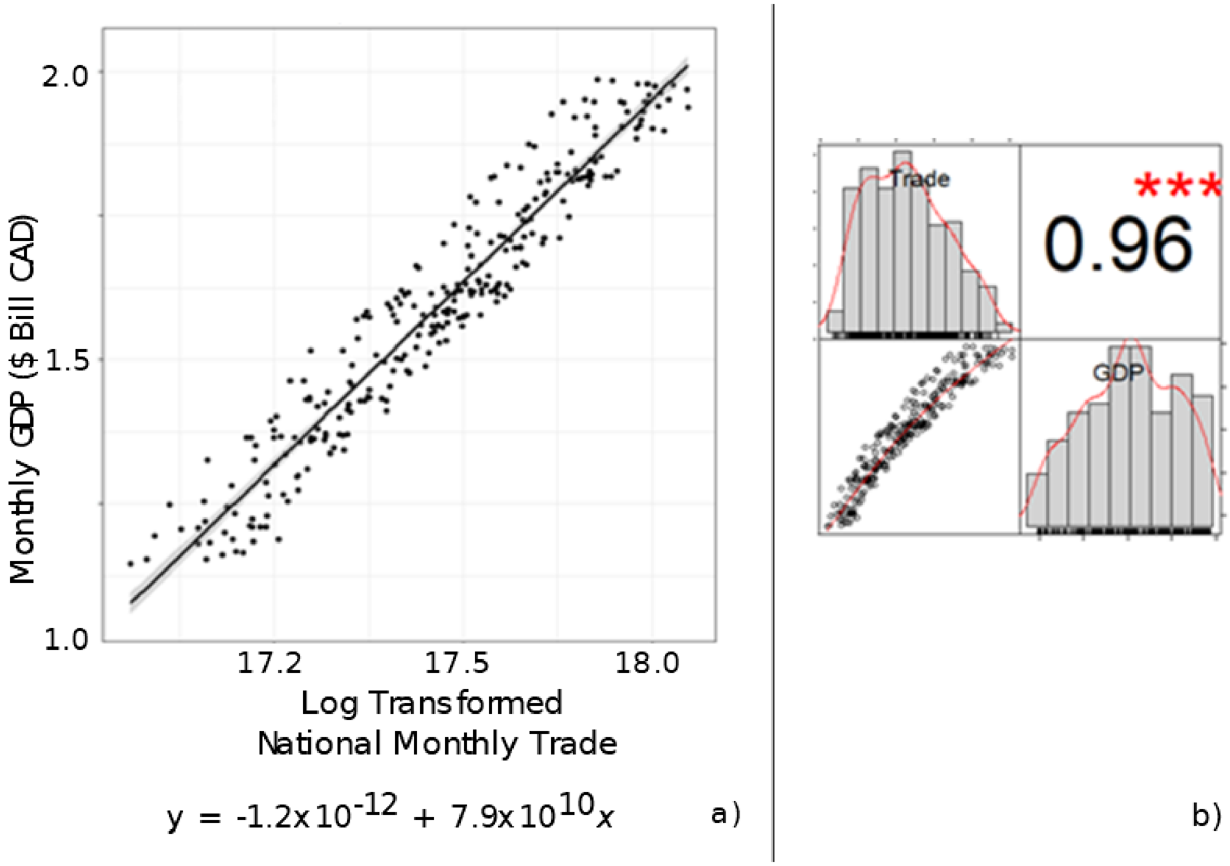

Supplementary Figure 5. The relationship between monthly GDP and trade data. The correlation is significant ($p<0.05$) with an $R^2>0.9$ using log-transformed trade data.

Supplementary Table 1. The explanation of variance for each of the climate-trade models by province. The LARS model is the only significant linear model and is thus the only linear model represented here. Linear models are significant (two-sided p-values, $p<0.05$), and a significance measure for the MARS model is also included (GR^2).

	**Provinces**									
**Models**	AB	BC	SK	MB	ONT	QUE	NL	PEI	NS	NB
LARS	32%	50%	21%	44%	49%	52%	49%	29%	43%	33%
RF (Annual)	19%	37%	3%	21%	27%	41%	22%	13%	29%	6%
Random Forest (Monthly)	35%	25%	21%	59%	42%	58%	44%	69%	50%	89%
RF w/Months	N/A	13%	24%	56%	45%	55%	49%	66%	41%	79%
MARS (2 deg)	55%	72%	68%	89%	58%	81%	61%	96%	67%	89%
GR^2	*0.38*	*0.58*	*0.33*	*0.65*	*0.47*	*0.76*	*0.46*	*0.71*	*0.56*	*0.82*
MARS (1 deg)	56%	76%	20%	79%	39%	55%	53%	41%	51%	91%
GR^2	*0.44*	*0.34*	*0.08*	*0.44*	*0.17*	*0.48*	*0.42*	*0.29*	*0.41*	*0.80*
MARS w/ Month	56%	71%	85%	89%	83%	81%	76%	96%	67%	86%
Max	***56%***	***76%***	***85%***	***89%***	***83%***	***81%***	***76%***	***96%***	***67%***	***91%***

Supplementary Table 2. The variables of greatest importance in the optimised MARS model. Total is for the entire binning of the optimized model. The seasonal variables are based on binning the data into seasons and optimising the MARS model under these circumstances. These models also did not explain as much of the entire year of data.

Province	Total	Winter	Spring	Summer	Fall
**BC**	Snow	Tmax	Snow	Tave	Tmin
**AB**	Tmax/Precip	Tmax	Tmax	Snow	Tmin
**SK**	Tmin/P/HDD/April	Precip	Tave	Tmin	Tmax
**MB**	Tmax/Snow/Precip	Tmin	Tmax	Tmax	Tmax
**ONT**	Snow/Precip/Nov	Tmin	Precip	CDD	Tmax
**QUE**	HDD	P	Tave	Precip	Tmax
**NB**	Tave/Snow/HDD	N/A	Tave	Tave	Tave
**NS**	HDD	N/A	N/A	N/A	N/A
**NL**	Tmax/Dec	N/A	N/A	N/A	N/A
**PEI**	Tmin/Snow/Precip/CDD	N/A	Tave/HDD	CDD	N/A

Supplementary Table 3. The economic trend projections based within the 30-year climate normal periods. The trend was assessed using the Mann-Kendall non-parametric statistical testing (two sided p-value).

	**Climate Normal 2020**		**Climate Normal 2050**		**Climate Normal 2080**	
**Province**	**Value**	**Significance**	**Value**	**Significance**	**Value**	**Significance**
BC	*-1.8*	$p=0.06$	*-3.07*	$p=0.02$	*-2.6*	$p=0.08$
AB	*0.004*	$p=0.0001$	*2.28*	$p=0.02$	*4.15*	$p=3.2^{e-5}$
SK	*-2.9*	$p=0.04$	*-4.6*	$p=3.1^{e-6}$	*-1.6*	$p=0.11$
MB	*7.9*	$p=0.002$	*9.96*	$p=2.2^{e-16}$	*6.3*	$p=3.3^{e-10}$
ONT	*10.2*	$p<2.2^{e-16}$	*6.1*	$p=8.7^{e-10}$	*4.5*	$p=4.7^{e-6}$
QUE	*11.8*	$p=2.2^{e-16}$	*12.1*	$p=2.2^{e-16}$	*8.8*	$p=2.2^{e-16}$
NB	*-5.75*	$p=9.2^{e-9}$	*-7.85*	$p=4.3^{e-15}$	*-5.6*	$p=1.6^{e-8}$
NL	*7.93*	$p=2.3^{e-15}$	*10.5*	$p=2.2^{e-16}$	*8.87*	$p=2.2^{e-16}$
NS	*10.6*	$p=2.2^{e-16}$	*11.0*	$p=2.2^{e-16}$	*6.3*	$p=2.9^{e-10}$
PEI	*-5.72*	$p=1.13^{e-8}$	*-7.44*	$p=1.02^{e-13}$	*-6.68*	$p=2.3^{e-11}$

Supplementary Table 4. The Mann-Kendall statistical testing of economic cycle trends (two-sided p-value). The Mann-Kendall statistic indicate the direction, magnitude, and significance of the cycle values produced by TIMESAT.

	Length		Amplitude	
Province	Trend	Significance	Trend	Significance
BC	0.04	p=0.03	2,812	$p=8.3^{e-11}$
AB	-0.03	p=0.0004	36,063	p=0.004
SK	0.03	p=0.0003	5,358	$p=2.2^{e-16}$
MB	0.01	p=0.16	4,007	p=0.0004
ONT	-0.005	$p=8.3^{e-11}$	-24,325	$p=2.2^{e-16}$
QUE	-0.004	$p=1.5^{e-9}$	-11,331	$p=2.2^{e-16}$
NB	0.03	p=0.002	2,149	$p=2.2^{e-16}$
NL	-0.02	$p=1.1^{e-7}$	388	$p=2.2^{e-16}$
NS	-0.01	$p=4.2^{e-16}$	-1,340	$p=2.2^{e-16}$
PEI	0.02	$p=1.2^{e-9}$	218	$p=2.2^{e-16}$
